# Report on Necrology

**Published:** 1892-09

**Authors:** 


### Report on Necrology.

The following testimonials and resolutions were presented, read and adopted at the recent meeting of the American Dental Association:

IN MEMORIAM—DR. JOHN ALLEN.

In the dispensation of an all-wise and over-ruling Providence Dr. John Allen, of New York, on the 8th day of March, 1892, at the age of eighty-two, passed from this to a higher and better life; having attained a fullness and ripeness of age beyond that of the common lot of men.

Dr. Allen stood as a representative man in the profession of his choice.

In the line to which he gave special attention he was the chief, and was so recognized not only in this, but in the countries of the world wherever prosthetic dentistry is known and practiced. He, it was, who brought to its present high state of perfection that variety of substitutes known as continuous gum dentures.

Though his chief attention and labor were devoted to this special work he was interested and took part in the various lines of thought and effort that were employed for the development, growth and establishment of dental science and art. He was ever ready to defend, and sought to elevate the profession to a higher plane of usefulness.

Dr. Allen was one of the organizers of the Ohio College of Dental Surgery, a professor and an efficient teacher in that institution.

In the subject of dental education he always manifested a warm interest. A writer of more than ordinary ability he has added many valuable contributions to the literature of the profession.

He was an active member of this association from almost the time of its organization and did much to promote its welfare. He was also a member of, and an active worker in, a number of other dental societies.

Dr. Allen was a man of purest character and highest integrity; one not only respected but loved by all who knew him; in manner most affable; in bearing dignified; in spirit gentle and sympathetic.

The loss of such a one is always an occasion of sadness and sorrow, but we have the consolation of the knowledge that his career was rounded, full and complete, and his death closed a life filled with good works for his fellowmen.

In view of the above,

*Resolved*, That we will ever cherish the memory of our departed brother and seek to establish and perpetuate the high principles that were so fully illustrated in his noble life.

*Resolved*, That the traits so pre-eminently characterizing the life of him we now commemorate are worthy, not only of our high regard, but most earnest emulation.

*Resolved*, That this testimonial be placed on a memorial page of the transactions of this body and a copy, properly engrossed, be sent to the family of the deceased; also that a copy be sent to the dental journals of this and other countries for publication.

---

### IN MEMORY OF C. A. KINGSBURY, M.D., D.D.S.

Within the last year Dr. Chas. A. Kingsbury was called from this to a higher life, in the seventy-second year of his age.

Dr. Kingsbury many years ago became identified with this association and retained his membership to the time of his death, and though he was not always present at its meetings, so highly was he esteemed by the membership of the body that it was a pleasure to all to have his name upon the roll of members.

Dr. Kingsbury entered the practice of the profession in 1839, in Philadelphia, and continued actively engaged in its pursuit during his life. He studied dentistry in Trenton, N. J. He was intimately acquainted with the leading men of the profession almost the whole of his professional career, and imbibed, in a large measure, the interest and enthusiasm of those men for dental science and art; indeed, that association, in a degree, shaped his professional life. He was familiar with all things that entered into the development and progress of dentistry for about fifty years. He was a man of liberal learning and broad culture; one whose sociability was a predominant characteristic. In his early life he was a teacher, and after many years practice of his profession he was for a time a successful teacher in one of the dental colleges in the city of his home. He was highly esteemed by all who knew him; he was a man of sterling characteristics, genial, kind and sympathetic in his association with his fellows. In his death, not only this association, but the entire profession loses another of the pioneers who was ever devoted to its interests, ever contributing of his resources to its up-building.

*Resolved*, That we will ever cherish the memory of our departed brother as one whom we delight to honor, and to emulate in his leading characteristics.

*Resolved*, That this statement and resolution be placed upon the memorial page of the proceedings of this body. That a copy, in proper form, be transmitted by the secretary to the family of the deceased, and that it be sent to the journals for publication.

